# Spatial Extent and Distribution of Ambient Airborne Particulate Matter (PM_2.5_) in Selected Land Use Sites in Nairobi, Kenya

**DOI:** 10.1155/2021/4258816

**Published:** 2021-11-13

**Authors:** Caroline Kiai, Christopher Kanali, Joseph Sang, Michael Gatari

**Affiliations:** ^1^Department of Soil, Water and Environmental Engineering, Jomo Kenyatta University of Agriculture and Technology, P.O. Box 62000-00200, Nairobi, Kenya; ^2^Department of Agricultural and Biosystems Engineering, Jomo Kenyatta University of Agriculture and Technology, P.O. Box 62000-00200, Nairobi, Kenya; ^3^Institute of Nuclear Science and Technology, College of Architecture and Engineering, University of Nairobi, P.O. Box 30197-00100, Nairobi, Kenya

## Abstract

Air pollution is one of the most important environmental and public health concerns worldwide. Urban air pollution has been increasing since the industrial revolution due to rapid industrialization, mushrooming of cities, and greater dependence on fossil fuels in urban centers. Particulate matter (PM) is considered to be one of the main aerosol pollutants that causes a significant adverse impact on human health. Low-cost air quality sensors have attracted attention recently to curb the lack of air quality data which is essential in assessing the health impacts of air pollutants and evaluating land use policies. This is mainly due to their lower cost in comparison to the conventional methods. The aim of this study was to assess the spatial extent and distribution of ambient airborne particulate matter with an aerodynamic diameter less than 2.5 *μ*m (PM_2.5_) in Nairobi City County. Seven sites were selected for monitoring based on the land use type: high- and low-density residential, industrial, agricultural, commercial, road transport, and forest reserve areas. Calibrated low-cost sensors and cyclone samplers were used to monitor PM_2.5_ concentration levels and gravimetric measurements for elemental composition of PM_2.5_, respectively. The sensor percentage accuracy for calibration ranged from 81.47% to 98.60%. The highest 24-hour average concentration of PM_2.5_ was observed in Viwandani, an industrial area (111.87 *μ*g/m³), and the lowest concentration at Karura (21.25 *μ*g/m³), a forested area. The results showed a daily variation in PM_2.5_ concentration levels with the peaks occurring in the morning and the evening due to variation in anthropogenic activities and the depth of the atmospheric boundary layer. Therefore, the study suggests that residents in different selected land use sites are exposed to varying levels of PM_2.5_ pollution on a regular basis, hence increasing the potential of causing long-term health effects.

## 1. Introduction

Clean air is a basic requirement for human well-being and health [1]. Air pollution poses a significant threat to human health, and it is largely attributed to the occurrence of high morbidity and mortality rates worldwide [2, 3]. Urban air pollution is of great concern in both developing and developed countries. Biomass use in both rural and urban areas has remained to be the main source of air pollution [4]. According to the WHO [5], more than 6 million premature deaths in 2012 occurred as a result of exposure to air pollution. However, more than 3 million of these deaths were attributed to ambient air pollution [4]. The ever-increasing population and increased volume of traffic in urban areas also have resulted in severe air pollution [6, 7]. Although there has been a significant air quality improvement in most high-income countries, middle- and low-income countries are still experiencing poor to very poor air quality.

Airborne particulate matter pollution is a major environmental risk factor with well-documented short- and long-term effects on human mortality and morbidity [8]. Epidemiological studies have reported a strong association between increased concentrations of inhalable particles and increased mortality. Falcon-Rodriguez et al. [9] and Turner et al. [10] illustrated that inhalation of particulate matter (PM) increases the prevalence of respiratory diseases, which include lung cancer and chronic obstructive pulmonary disease. PM exists in the atmosphere as either liquid or solid particles with varying sizes and compositions [11], Harrison 2 et al. [12]. The size and distribution of particles are characterized by ultrafine, fine, or coarse particles, each with an aerodynamic diameter equal to or less than 0.1, 2.5, and 10 *μ*m, respectively. Particles with a diameter equal to or less than 10 and 2.5 *μ*m, commonly referred to as PM_10_ and PM_2.5_, respectively, are of special importance as they can reach the upper and lower portions of the respiratory tract of exposed individuals and can cause cardiovascular and respiratory illness [8, 13].

According to Shilenje et al. [14], Nairobi County has undergone a significant land use/cover change transformation principally occasioned by increasing numbers of residential estates, city expansion, industrialization, and a rising human population. According to a report by the JICA, Nairobi is ranked as the most industrialized urban center in East and Central Africa [15]. A total of 338 industries were registered in 2004 with the Directorate of Occupational Health and Safety (DOHS) [15]. Previous studies in Nairobi on air pollution have specifically determined the pollution levels on industries, major roads, and residential and commercial areas separately [16–20]. However, a comparison of the levels in the different land uses is important in an attempt to quantify the effect of urbanization on the environment.

As one of the developing countries, Kenya experiences serious environmental challenges concerning air quality deterioration, water pollution, noise pollution, and soil contamination. Most research has been done regarding water pollution and degradation of soil, but little attention has been given to air quality deterioration. This has led to limited information on air pollution within the urban centers. Therefore it is difficult to assess the impacts of urban development and increase in urban population. This study, therefore, aimed to assess the spatial extent and distribution of ambient airborne particulate matter (PM_2.5_). This assessment is important to decision-makers, planners, and regulatory bodies in the sustainable management of air quality in urban centers.

## 2. Materials and Methods

### 2.1. Study Area

**Figure 1:**
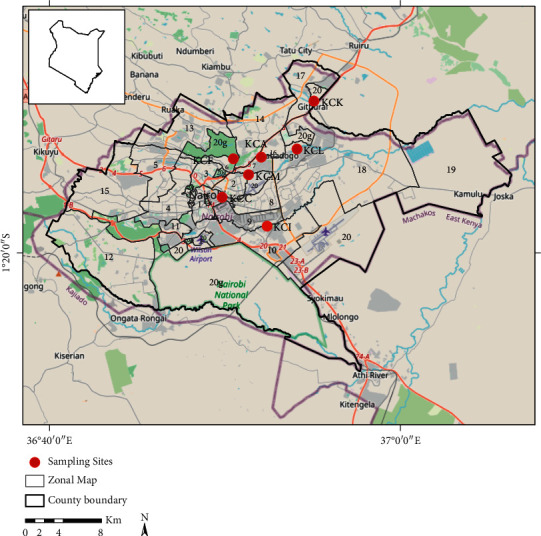
Location of selected sampling sites and their respective zone [9] for PM_2.5_ mass concentration level monitoring in Nairobi City County, Kenya.

This study was conducted in selected land use sites within Nairobi City County, Kenya. Nairobi is Kenya's capital city, located at 1° 9′S, 1° 28′S and 36° 4′E, 37° 10′E (Figure 1). The city experiences four major seasons: warm dry season (December–February), the long rainy season (March–May), the cool and dry season (June–October), and the short rainy season (October–November) [21]. The altitude varies between 1600 m and 1850 m above the sea level and occupies an area of about 696 km^2^. According to KNBS [22], the population of Nairobi is estimated to be about 4.4 million people. Rapid population growth of about 4.6% per year has led to high congestion. The city has a population density of 3080 persons per square km compared to 50 persons per square km countrywide [23]. Temperatures generally vary from 12°C during the cold season to 29°C during the warm and dry season from December to March.

The monitoring points selected for this study were Viwandani (KCI), Mathare (KCM), Karura (KCF), CBD (KCC), Lucky Summer (KCL), Allsops (KCA), and Kahawa (KCK) (Table 1). The sites were selected using nonprobability sampling technique, which involved selecting the sites based on the land use activity within the relevant areas that represented the entire study area.

### 2.2. Calibration of Low-Cost Sensors

This study was conducted using PMS7003 sensors by Plantower and cyclone samplers (BGI 400S) to monitor PM_2.5_ mass concentration levels. PMS7003 sensors were chosen because of their low-cost ($30), previous versions of PMS sensors (PMS1003 and PMS5003) were evaluated, and their performances were propitious for monitoring PM_2.5_ [24]. The sensors have a measurement chamber with a set of focusing lenses, light-emitting diode, and photodiode detector. They use the light scattering principle to measure the size distribution of fine particles using Mie theory [25]. Eight sensors were calibrated before deployment for four consecutive days (86 hours) from 4^th^ to 7^th^ December 2020 according to the procedure adopted by Pope et al. [26]. This involved colocating the sensors with a standard Andersen dichotomous impactor (Sierra Instruments Inc., USA) [21] which is located at the University of Nairobi (UON) 17 m above the ground away from traffic sources. The impactor has a sampler that segregates the stream of air that passes through it into coarse and fine portions that are filtered on preweighed Teflon filters of a diameter of 37 mm and a 2 *μ*m pore size. Filters were replaced after every 24 hours and weighed in the laboratory. The 24-hour average mass concentration of PM_2.5_ was then obtained by dividing sample mass after accounting for all the uncertainties over the total volume. The uncertainties were estimated to be 10% instrument error, 25% weighing, and 7% sampling error [26]. A comparison of the mass concentration obtained from the optical method and gravimetric method was made using the following equation to obtain the correction factor for each sensor that was applied to all the data collected from the different sites:

(1) $$\begin{array}{c} \text{CF}=\frac{\text{GMC}}{\text{OMC}}. \end{array}$$

CF is the correction factor (scaling factor), GMC is the average gravimetric mass concentration, and OMC is the average optical mass concentration.

Accuracy assessment of the sensors was obtained using equation (2) that was adopted by Liu et al. [27] to evaluate the degree of closeness between the values measured by the sensor and the reference:

(2) $$\begin{array}{c} A\%=100-\;\frac{\left|X-R\right|}{R}\times 100. \end{array}$$

Here, *A*% is the accuracy percentage, *X* is the average concentrations measured by the sensor for the entire calibration period, and *R* is the average concentration measured by the reference monitoring station.

### 2.3. Sensor-Based Monitoring

The calibrated sensors were deployed one sensor per site to the seven sites with different land use types (Table 1). They were mounted to monitor ambient PM_2.5_ at heights ranging from 1.5 m to 2.5 m above the ground level which is close to human breathing height. The sensors were programmed to log the concentration levels after every 1 minute continuously. Monitoring was done from 7^th^ January to 29^th^ March 2021. The data collected were analyzed using *R* statistical software version 4.1.0 to obtain hourly and daily average concentrations of PM_2.5_. The Kriging Geostatistical method was used to interpolate the hourly averages point data of PM_2.5_ in ArcGIS software version 10.2 because it assumes that the direction or distance between sample points reveals a spatial correlation used while explaining the variation in the surface [28]. The general formula is formed as a weighted sum of the data and is given by

(3) $$\begin{array}{c} Z\left(\mathrm{so}\right)=\sum_{i=1}^{n}ƛiZ\left(\text{si}\right)\;. \end{array}$$

Here, *n* is the number of values measured, *Z* (si) is the value measured at the ith location, and *λ*_*i*_ is the weight for the measured value that is unknown at the ith location and so is the prediction location.

### 2.4. Gravimetric-Based Monitoring

Gravimetric-based monitoring was done for a period of 1 month to collect air samples from the selected land use sites for elemental composition of PM_2.5_. During this period, three samples were collected per site on weekdays for nonconsecutive days following the procedure that was used by Maina et al. [20]. Sampling was done using a BGI 400S personal sampling pumps with a cyclone. The personal sampler is a cyclone sampler with a geometry that allows only particles with an aerodynamic diameter of less than the required measurement to be deposited on the filter media. The principle of operation for the cyclone sampler is the theory of particle inertia to collect/select particles of a particular size range [29].

Teflon filters of a diameter of 37 mm and a 2 *μ*m pore size were used to collect PM_2.5_. Clean Teflon filters were then loaded onto the filter holder. The sampler used has a rechargeable battery that ran for 8 hours. This made it possible for PM_2.5_ sample collection even at remote sites. Preweighed filters were loaded into cassettes, which were then connected into the nozzle and placed in a sampling position. Samples were collected for 8 hours (8 a.m. to 4 p.m.) after which the filter cassettes were offloaded and sealed carefully in an air tight Petri dishes and stored in a clean environment to avoid the contamination of the collected samples. The flow rate was measured when the clean Teflon filters were loaded and after the sampling was done using a field rotameter. The rotameter was calibrated at different flow rates in the laboratory using a flow meter to facilitate a calibration graph for use in establishing correct average sample flow after the field sample collection [30]. PM_2.5_ concentrations were calculated by the difference between the initial and the final weighting divided by the total volume of air that passed through the filter [31].

### 2.5. Elemental Composition of PM_2.5_

The elemental composition of PM_2.5_ was determined by a nondestructive method using an energy dispersive X-ray fluorescence (EDXRF) spectroscopy at the Institute of Nuclear Science laboratory at the University of Nairobi. The air samples collected from the gravimetric-based monitoring were analyzed for K, Ca, Ti, Mn, Fe, Ni, Cu, Zn, Ca, As, Br, Zr, and Pb. The spectrometer is made up of a Si (Li) detector that is used in detecting the spectral line characteristic, a sample chamber, and an emission energy analyzer. The filters were subjected to a current of 80 *μ*A and a voltage of 30 kV for a period of 1500 seconds. The spectra were analyzed for quantitative analysis using the AXIL program following the procedure outlined by [21] to determine the concentrations in mass per unit area for thin film and later converted to mass per unit volume (concentrations in ng/m³).

## 3. Results and Discussion

### 3.1. Calibration of the Sensor Results

**Figure 2:**
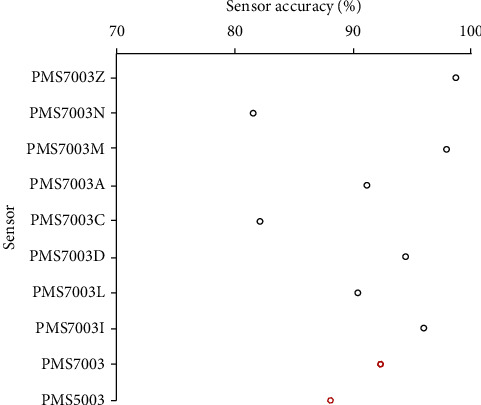
Sensor accuracies in percentage obtained in this study in comparison with accuracy percentages reported in a study that used a similar sensor model. Red dots represent accuracy values reported by Badura et al. [24].

The results of the calibration of the eight sensors are as shown in Table 2 and Figure 2. Table 2 shows the average concentrations of PM_2.5_ from the standard measurements and sensor-based measurement and the scaling factors for each sensor. The scaling factors ranged from 1.09 ± 0.58 to 1.46 ± 0.93. Pope et al. [26] reported a scaling factor of 1.63 after performing a similar procedure at the same site with similar standard equipment. The variation in the scaling factors could be attributed to the variation in the sensor model used since they were using OPC-N2 sensors.

Figure 2 shows the accuracy percentage of the different PMS7003 sensors. The accuracies were determined using equation (2), and they ranged from 81.47% to 98.60%. Badura et al. [24] obtained similar accuracies (Figure 2) for the PMS7003 sensor model. The earlier versions of Plantower sensors (PMS5003 and PMS3001) were also reported to have good and higher repeatability [24, 32]. This showed that the sensors' level of accuracy is high and can be used to monitor PM_2.5_.

### 3.2. Sensor-Based Monitoring Average Concentrations

**Figure 3:**
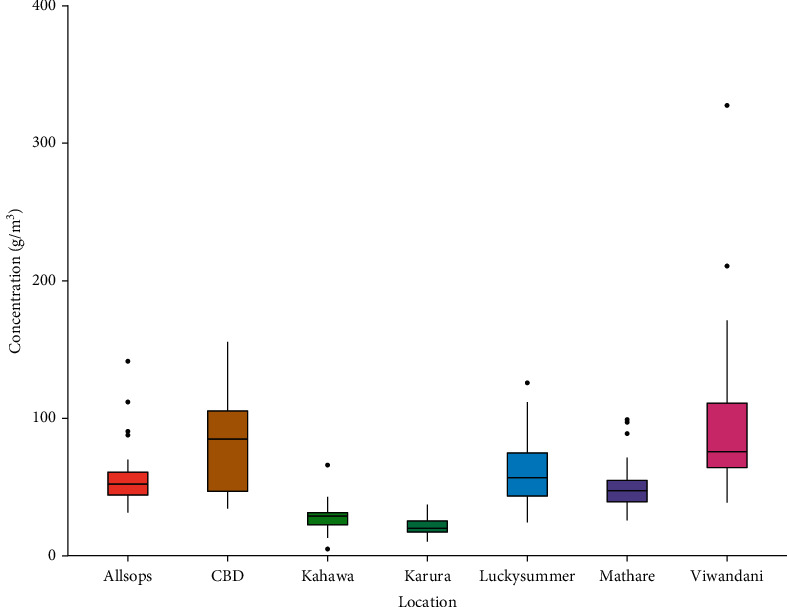
24-hour mean distribution of PM_2.5_ mass concentration (*μ*g/m³) in the selected land use sites in Nairobi City County.

Figure 3 shows the distribution of PM_2.5_ mass concentrations of the sites selected within Nairobi. The 24-hour mean for all the sites exceeded the WHO recommended guideline for PM_2.5_ (25 *μ*g/m³) except for one site, which is a background site that is forested (Table 1). The box plot distribution (Figure 3) in all the sites indicates that, in most cases, the residents are exposed to PM_2.5_ pollution. The values for the average daily mass concentration levels ranged from 21.25 *μ*g/m³ at the urban background to 111.87 *μ*g/m³ at an industrial site. The Viwandani site (Figure 1) had the highest average concentration levels of PM_2.5_ of 111.87 *μ*g/m³ for the three-month period of monitoring. This average is higher than the ambient air quality tolerance limit recommended by NEMA for an industrial area in Kenya of 75 *μ*g/m³ [33]. Many contributions were attributed to the surrounding industries, and part of them were from waste burning and indoor air.

The minimum and the maximum values recorded for the 24-hour mean at Viwandani were 38.75 *μ*g/m³ and 513.84 *μ*g/m³, respectively. Mutahi et al. [30] reported a mean value of PM_2.5_ of 166 *μ*g/m³ at Viwandani, which is slightly above the average value reported in this study. This difference might have been due to the differences in sampling seasons and the 12-hour sampling time per day by Mutahi et al. [30]. However, a study by Shilenje et al. [14] showed low concentrations of PM_2.5_ in the industrial area, which is largely attributed to the variations in meteorological parameters [34] during the monitoring period, precipitation of 0.52 mm/hr [14].

CBD site (Figure 1) had the second-highest 24-hour mean concentration of 82.56 *μ*g/m³ with the values ranging from 34.42 *μ*g/m³ to 155.58 *μ*g/m³. These values are higher than the WHO recommended standard of 25 *μ*g/m³. The site is characterized by high pedestrian and vehicular traffic, retail shops, street vendors, and other businesses. Kinney et al. [17] reported values ranging from 75.6 to 98.1 *μ*g/m³ for 11-hour averages at different roundabouts within the CBD: Ronald Ngala, Tom Mboya, and River Road. Lucky Summer had a 24-hour average value of 60.00 *μ*g/m³, which is more than twice the Air Quality Guidelines (AQI) recommended by WHO. This is to a large extent explained by the unpaved roads [35, 36], the large nearby dumpsite [37], and industrial sources from Babadogo [38]. Mathare site (Figure 1) had a 24-hour average of 50.07 *μ*g/m³ with values ranging from 2.63 to 270.38 *μ*g/m³. Ngo et al. [39] reported an 8-hour average of 62 *μ*g/m³. The results from this study are slightly lower than those reported by Ngo et al. [38]. This could be attributed to the differences in the sampling period since the study sampled PM_2.5_ levels for 8 hours during daytime only, and there are fewer aerosol concentrations at night [40], which reduces the average 24-hour mean value. Kahawa site (Figure 1), on the other side, had a 24-hour mean of 28.37 *μ*g/m³, which is slightly above the WHO recommended standard for PM_2.5_. The low concentrations could be attributed to the surrounding vegetation cover around the site that could have reduced pollution levels [17].

**Figure 4:**
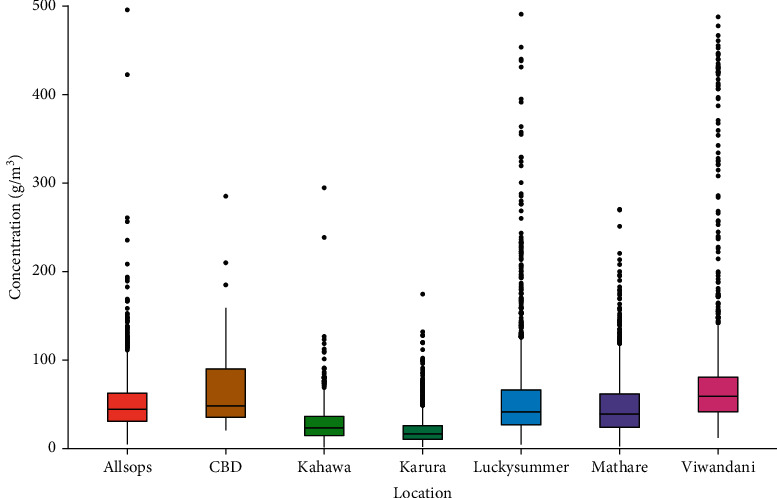
Hourly distribution of PM_2.5_ mass concentration (*μ*g/m³) in the selected land use sites in Nairobi City County.

The lowest PM_2.5_ concentrations were observed at the Karura site (Figure 1). The 24-hour mean for the study period was 21.25 *μ*g/m³, which is lower than the 25 *μ*g/m³ recommended by WHO. From the analysis, it was observed that 72% of the PM_2.5_ measurements were above 25 *μ*g/m³ and 28% were below 25 *μ*g/m³. This shows that PM_2.5_ mass concentrations are below the WHO recommended standard on most of the days in Karura, which is highly attributed to the fact that the tree cover in this site reduces PM_2.5_ pollution. However, the hourly distribution of PM_2.5_ as shown in Figure 4 for this site indicates that some hours have much higher concentrations with values ranging from 1.92 and 174.57 *μ*g/m³, respectively. These values are higher than the values reported for the 24-hour mean for background sites [17, 20, 26]. Several studies have shown a significant reduction of air pollutants in areas with vegetation cover [2, 41, 42].

**Figure 5:**
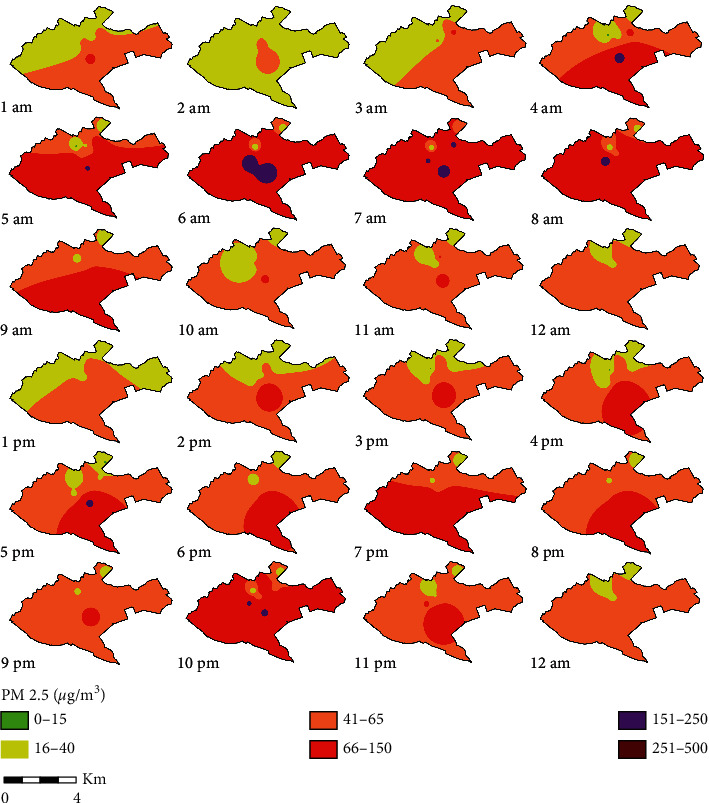
Spatial distribution of mean hourly PM_2.5_ mass concentration in Nairobi City County.

Figure 5 shows the spatial distribution of PM_2.5_ in Nairobi City County. The color codes adopted for this are as stipulated by US EPA's breakpoint for PM_2.5_ and Air Quality Index (AQI) [43]. The green color is good, yellow is moderate, orange is unhealthy for sensitive groups, red is unhealthy, purple is very unhealthy, and maroon is hazardous. The results show that there is a trend of peak during morning hours from 0500 to 0900 hours and evening peak that starts from 1600 to 2200 hours. From 1100 to 1500 hours, the larger part of Nairobi is under moderate AQI, and some parts around Kahawa, Westlands, and Dagoretti have PM_2.5_ levels that are unhealthy for sensitive groups. The area around the industrial area (Viwandani) shows that the area is under PM_2.5_ pollution that is unhealthy to the residents most times of the day except for a few times around midnight to 0300 hours. A study by Yadav et al. [40] observed similar peaks of PM_2.5_, which occurred between 0700–1000 and 1900–2300 hours. In residential areas with informal settlements, much of the contribution to PM_2.5_ in the evening is due to increased cooking activities. Other studies have also observed similar trends [13, 44–47]. There is a decrease in PM_2.5_ concentration levels at mid-morning (10 a.m.) to late afternoon/early evening (4 p.m.) due to the increase in the atmospheric boundary layer and low road traffic flows [45].

### 3.3. Gravimetric-Based PM_2.5_ Concentration Levels and Elemental Composition

**Figure 6:**
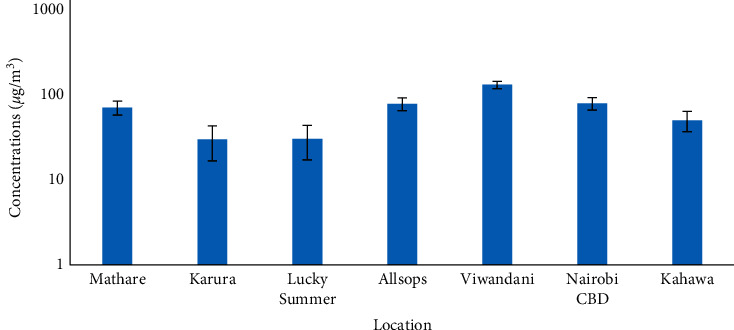
The average 8-hour average PM_2.5_ concentration levels in the selected land use sites within Nairobi City County.

**Figure 7:**
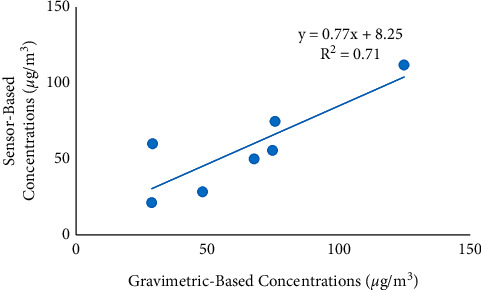
Comparison between the average sensor-based monitoring and gravimetric-based using cyclone pump samplers (BGI 400S) concentration.

The 8-hour average concentrations of PM_2.5_ obtained from weighing the filters and applying equation (1) are shown in Figure 6. The three-day nonconsecutive sampling using cyclone samplers showed that Viwandani had the highest average PM_2.5_ concentrations (124.87 *μ*g/m^3^) and Karura had the lowest (28.82 *μ*g/m^3^). The results show a high and positive correlation between gravimetric-based and sensor-based measurements as the coefficient of determination (*R*^2^) was 0.71. In addition, there is low significant variability between data measured using the cyclone samplers and Plantower PMS7003 sensors (Figure 7). The 29% variability could be due to filter handling [26] and the sampling period which tends to reduce the average concentrations when done for longer periods [30].

**Figure 8:**
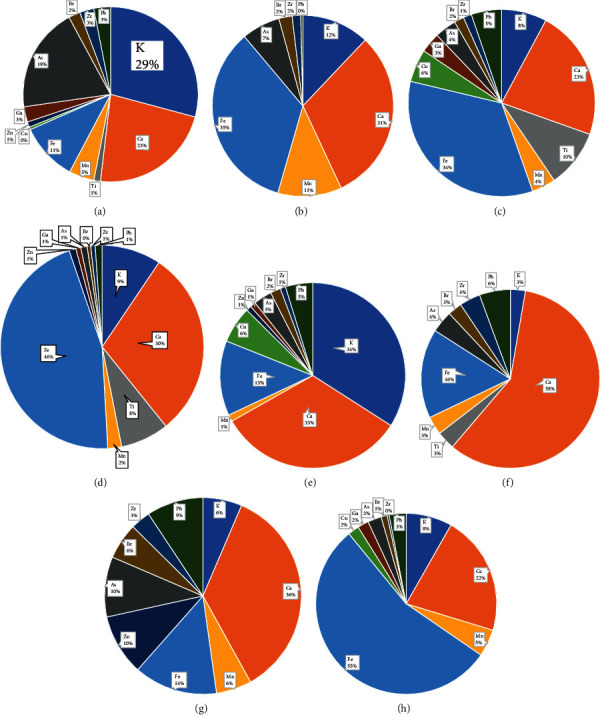
The relative percentage concentrations of major elements in PM_2.5_ collected in the filter samples using Cyclone pump samplers (BGI 400S) for selected land use sites in Nairobi City County for an 8-hour period: (a) KCM, (b) KCL, (c) KCA, (d) KCI, (e) KCC, (f) KCK, (g) KCF, and (h) KCU.

Figure 8 shows the relative percentage distribution of elemental composition in PM_2.5_ samples for each site. The pie charts were constructed from the average concentration values for the selected elements in PM_2.5_. The number of elements detected in PM_2.5_ filter samples were 13: K, Ca, Ti, Mn, Fe, Ni, Cu, Zn, Ca, As, Br, Zr, and Pb. The most abundant elements are Fe, K, and Ca, largely attributed to the natural resources that include Earth's soil, crust, and suspended dust from unpaved roads [48, 49].

The presence of Zn, Pb, As, K, Mn, and Cu suggests the presence of both anthropogenic and natural sources [11]. Natural sources mainly include Earth's crustal dust, and the anthropogenic include emissions from combustion of fossil fuels, car tires, and breaks [31]. KCF and KCU (Table 1) had lower concentrations of elements compared to all the other sites, and KCI had higher elemental concentrations, with Fe, Ca, and K having higher concentration levels because KCF is a forested area and the elements here are likely to have a natural source mainly soils. The forest of trees and scrubs around the area decreases the amount of dust that emanates from the ground [31]. The concentrations of all the elements ranged from a minimum of 6.79 to 44,668.42 ng/m^3^. According to Gordon [11], the sources of K are highly linked to wood combustion, soils, incinerators, and lime kilns. Zn and Mn elements indicate that the source could be from metal industries [50, 51]. Elements such as Ca, Ti, Fe, and Mn are classified as crustal elements and are characterized by resuspended dust particles related to traffic and unpaved roads [38].

Table 3 shows the statistical correlation coefficient *r* of the elements in PM_2.5_ samples. The results indicate that there are significant correlations between PM_2.5_ concentration levels and K, Ca, and Ti.

Mn, Fe, Zn, Ga, Br, Zr, and Pb coefficients range from 0.65 to 0.88. This shows that there are positive correlations between most of the elements in PM_2.5_ which suggests that they originate from more common/similar sources. The elements with (*r*) that is more than 0.5 shows that there is a strong relationship between the elements [20]. High correlations were observed between K, Ca, Ti, Br, Ga, and Zn. The high correlation between K, Ca, Ti, and Ca is predominantly thought to originate from soils that are generated from soil emissions through transportation [31].

## 4. Conclusion

This study showed that the sensor accuracies ranged from 81.47% to 98.60%, which indicates that low-cost sensors can be used to monitor air quality provided they are calibrated before deployment. They can provide useful information that can be used by urban planners, decision-makers, private sector, government, and the public in general.

The results of the distribution of PM_2.5_ in the selected land use sites ranged from a daily mean of 21.25 *μ*g/m^3^ to 111.87 *μ*g/m^3^. The observed 24-hour mean for all the sites was above the WHO recommended standard of 25 *μ*g/m^3^ except for the Karura site (Figure 1), which is a forest reserve (21.25 *μ*g/m^3^). This implies that many residents in Nairobi are on regular occasions exposed to PM_2.5_ pollution, which has the potential of causing a long-term impact on health. However, vegetation cover has a great potential of reducing PM_2.5_ pollution and toxic elements hence the need to sensitize the public on greening the city in an effort to improve urban air quality.

## Figures and Tables

**Table 1:** Description of the location of the selected sites, sampling site codes, and prevailing land use according to the Nairobi zonal map.

Site name	Code	Latitude	Longitude	Description
Viwandani	KCI	−1.3086	36.8737	Main industrial area
Mathare	KCM	−1.2617	36.8561	High-density residential flats (informal settlement)
Nairobi CBD	KCC	−1.2823	36.8310	Commercial/residential/light industry
Karura	KCF	−1.2470	36.8417	Park/forest/recreation
Lucky Summer	KCL	−1.2383	36.9019	Residential mixed development/industrial
Allsops	KCA	−1.2455	36.8681	Low-density residential (site was 15 m away from Thika Superhighway)
Kahawa	KCK	−1.1943	36.9181	Agricultural, residential mixed development
University of Nairobi	KCU	−1.2794	36.8163	Location of the Andersen Dichotomous impactor, mounted 17 m above the ground level

**Table 2:** Average standard gravimetric measurement and sensor-based averages of different PMS7005 sensors and their scaling factors.

Average gravimetric concentrations (*μ*g/m³)	Sensors	Average sensor concentrations (*μ*g/m³)	Scaling factor
20.70 ± 4.12	PMS7003Z	16.23 ± 9.27	1.28 ± 0.74
	PMS7003N	18.92 ± 10.62	1.09 ± 0.58
	PMS7003M	15.78 ± 8.47	1.31 ± 0.78
	PMS7003A	18.37 ± 9.80	1.13 ± 0.60
	PMS7003C	14.16 ± 3.61	1.46 ± 0.93
	PMS7003D	17.60 ± 10.13	1.18 ± 0.65
	PMS7003L	18.87 ± 9.17	1.10 ± 0.58
	PMS7003I	17.33 ± 11.13	1.19 ± 0.66

Note: the letters *Z*, N, a, K, A, C, D, L, and I are used to differentiate the sensors since they are the same model (PMS7003).

**Table 3:** The correlation coefficient *r* between average concentrations of the elemental composition of PM_2.5_ in selected land use sites in Nairobi City County.

	K	Ca	Ti	Mn	Fe	Cu	Zn	As	Ga	Br	Zr	Pb	PM_2.5_
K	1												
Ca	0.699	1											
Ti	0.5935	0.9343	1										
Mn	0.5861	0.8092	0.8195	1									
Fe	0.6431	0.9481	0.9897	0.8468	1								
Cu	0.3319	−0.1172	−0.1784	−0.3386	−0.1831	1							
Zn	0.686	0.842	0.8678	0.6963	0.8907	−0.1798	1						
As	0.4538	0.052	−0.0165	0.3042	−0.01	−0.1177	0.0964	1					
Ga	0.7395	0.617	0.7064	0.6573	0.6698	0.123	0.6114	0.5384	1				
Br	0.7406	0.738	0.54	0.6403	0.5527	0.0695	0.5699	0.5073	0.5876	1			
Zr	0.5212	0.7385	0.5783	0.6522	0.5615	−0.256	0.4902	0.5051	0.5555	0.8911	1		
Pb	0.6436	0.6457	0.4841	0.2798	0.45	0.4552	0.4347	0.1783	0.5416	0.8079	0.6904	1	
PM_2.5_	0.8349	0.8407	0.8288	0.6827	0.8099	0.2824	0.7236	0.2565	0.8793	0.7522	0.6452	0.7964	1

## Data Availability

The data used to support the findings of this study are available from the corresponding author upon request.
